# A Virtual Environment to Improve the Detection of Oral-Facial Malfunction in Children with Cerebral Palsy

**DOI:** 10.3390/s16040444

**Published:** 2016-03-26

**Authors:** María-Luisa Martín-Ruiz, Nuria Máximo-Bocanegra, Laura Luna-Oliva

**Affiliations:** 1 Department of Telematic and Electronic Engineering, Technical University of Madrid, Madrid 28031, Spain; 2 Department of Physiotherapy, Occupational Therapy, Rehabilitation and Physical Medicine, Faculty of Health Sciences Rey Juan Carlos University, Alcorcón, Madrid 28922, Spain; nuria.maximo@urjc.es (N.M.-B.); laura.luna@urjc.es (L.L.-O.)

**Keywords:** serious games, inclusive education, detection, virtual therapeutic environment, virtual reality

## Abstract

The importance of an early rehabilitation process in children with cerebral palsy (CP) is widely recognized. On the one hand, new and useful treatment tools such as rehabilitation systems based on interactive technologies have appeared for rehabilitation of gross motor movements. On the other hand, from the therapeutic point of view, performing rehabilitation exercises with the facial muscles can improve the swallowing process, the facial expression through the management of muscles in the face, and even the speech of children with cerebral palsy. However, it is difficult to find interactive games to improve the detection and evaluation of oral-facial musculature dysfunctions in children with CP. This paper describes a framework based on strategies developed for interactive serious games that is created both for typically developed children and children with disabilities. Four interactive games are the core of a Virtual Environment called SONRIE. This paper demonstrates the benefits of SONRIE to monitor children’s oral-facial difficulties. The next steps will focus on the validation of SONRIE to carry out the rehabilitation process of oral-facial musculature in children with cerebral palsy.

## 1. Early Detection and Intervention in Children with Cerebral Palsy

Cerebral palsy (CP) is a continuous but non-progressive motion/posture, motor function disorder resulting from an injury in the developing brain primarily damaging the areas responsible for the postural control [1,2,3]. Motor disorders in people with CP are often accompanied by disturbances of sense, perception, cognition, communication, as well as behavioral issues, epilepsy and secondary musculoskeletal problems [4]. CP is the most common cause of severe disability in childhood [5], and can also involve physical, cognitive, emotional and social difficulties [6,7].

The different impairments that affect children with CP make it very hard, or even impossible, to perform many daily activities (getting dressed, personal hygiene, eating, going to school, and even walking and talking). Although the physical and motor difficulties and changes are often the most obvious, cognitive, emotional and behavior impairments are present in 50% to 75% of cases [8]. The effect of this whole set of problems goes beyond the individual; it can also affect the family and, furthermore society.

A very important aspect of this disorder, which must be considered in addition to the abovementioned considerations, is that it affects a person as they are maturing, before developmental milestones have been reached. The acquisition of these milestones enables an individual’s participation in society, which, for the World Health Organization (WHO), would mean reaching a state of health. The injuries that cause CP may occur during pregnancy, childbirth, or in the first few years of life, and may be derived from several different causes, some of which are still unknown. Supportive treatments, medications, and surgery can help many individuals improve their motor skills and ability to communicate with the world [9].

According to the Surveillance of Cerebral Palsy in Europe (SCPE), a network made up of 20 European countries, the incidence of CP is currently 2 to 3 per 1000 births [3]. However, this number is rising, due to an increase in new cases and the rising life expectancy of those affected [5,10]. This rate goes up to 40–100 per 1000 live births among babies born very prematurely or with a very low birth weight [11]. Rates appear to be similar in both the developing and developed world [12]. The rate is higher in males than in females; in Europe it is 1.3 times more common in males [13].

It is, therefore, a primary healthcare problem, and its care must be encompassed in an integral care model, based on a set of interventions focused on the childhood population and its environment [14]. These interventions must have the objective of responding to the needs of children with developmental disorders, or at risk of acquiring them, from both the healthcare and the psychological and social perspectives to strive for the greatest degrees of personal autonomy and family, school and social integration.

The importance of therapeutic intervention in CP is well accepted, hence new tools have been developed to create contexts and situations capable of facilitating the interaction between the individual and the environment [1,15]. In fact, the search for solutions that provide benefits for patients treated with Virtual Reality (VR)-based systems has included devices that were initially designed for other uses (Xbox 360, Kinect, Wii), but that have proven useful when applied in the medical world [16,17]. In addition, the low cost of these technologies, compared to specialized hardware, makes them even more attractive. The term VR was introduced by Jaron Lanier in 1986. However, due to the progress of technology, VR has undergone several transformations since then [18]. VR is classified according to the degree of immersion the user experiences. According to Burdea and Coiffet [19], the term immersion is the voluntary act of ignoring the different stimuli that make users perceive the experience as not real, allowing the system to hold users’ full attention and concentration. VR has been widely used in the field of medicine, since it can present challenging situations within a safe environment, while maintaining experimental control of the mediation and presentation of stimuli [20]. The first applications of this kind gave way to a series of psychosocial interventions for the treatment of phobias [21]. Later, its use was expanded into other areas, such as surgical training and neurological rehabilitation, among others [18]. In neuro-rehabilitation, VR is used as a tool for rehabilitation and evaluation in programs for training balance, posture and walking, activation of functions in the upper limbs, exercise and pain tolerance therapies, evaluation of daily activities, and evaluation of visual impairment after a stroke [22,23].

Reviewing the literature, we found the work of Luna *et al.*, presenting a study that had the objective of evaluating the utility of a video game system based on VR technology: the Xbox 360 Kinect [24]. The system developed has the objective of aiding the conventional rehabilitation treatment that is traditionally prescribed to children with CP. The study included 11 children whose motor skills were measured, specifically, balance, speed when walking, running and jumping, and fine motor skills. The participants underwent the treatment with the Xbox 360 Kinect for eight weeks. The statistical study applied the Wilcoxon test [25], which showed significant differences before and after the treatment for all the values measured. In addition, the evolution of these children was monitored for a year, obtaining stable results carried out from the previous evaluation, which proves that this therapy has persistent long-term positive effects.

The study performed by Sharan *et al.* is important because it was the first research to use VR-based therapy (Wii Fit) for post-operatory rehabilitation in children with CP [26]. The main conclusion of this study was that balance improved significantly in children who used this technology during their rehabilitation process. Their participation level, motivation, cooperation and satisfaction during therapy also improved.

Other studies of children with neural development problems are focused on autism. The main feature of this pathology is that these children have severe and generalized deficiencies in their social interactions and communication. The work of Blum-Dimaya *et al.* was focused on showing four children with autism how to use the video game Guitar Hero II [27]. In addition to the game, the children were given an activity chronogram that taught them how to work with the game. As a result, all four participants managed to learn how to play Guitar Hero II. These results were very beneficial, since the musical sounds also functioned as stimuli to enable verbal and non-verbal communication. This is possible as music acts at a neural level.

Finally, we must include the results obtained during a systematic review of the literature. The work of Wang and Reid in 2011 contains a systematic review of the existing literature exploring VR systems used as treatment tools that are meant to deal with the main deficiencies of children with attention deficit disorder with and without hyperactivity (ADHD), autism and CP [28]. In this paper, they worked with 20 articles, for which they considered two aspects; the first was the analysis of general benefits of VR in the context of pediatric rehabilitation. The second was the testing of the results obtained in all the studies. An important result of this review was that simple VR systems used at home can help increase adherence to treatment programs in children, because these systems increase the degree of participation in activities and reduce the need to go to rehabilitation centers where conventional therapy is received. Furthermore, they found that the studies performed on children with CP used VR in combination with treadmill training, and the results were that it enabled the rehabilitation of the lower limbs. This fact allowed the children to achieve full immersion in the video game, bringing them into the virtual environment.

The meta-analysis performed by Chen *et al.* intended to systematically evaluate the effect of VR in achieving mobility in the upper limbs, associating its effects to the importance of adhesion to treatment [29]. The conclusion of this review was that VR is a viable tool for improving motor function in the upper limbs of children with CP. Besides, it stated that it is necessary to continue this line of investigation to provide a conclusive recommendation.

In this paper, we present a virtual environment to improve the detection and evaluation of oral-facial musculature dysfunctions in children with CP. This article is organized as follows. After this introduction, in Section 2, Section 3 and Section 4 we describe the Sistema de terapia basadO en KiNect paRa nIños con parálisis cErebral (Virtual Environment using Kinect for children with CP, known by its Spanish acronym SONRIE—“smile” in English), as an intervention and detection system. Section 5 and Section 6 present a verification process performed with both typically developing children and children with CP. Finally, we discuss the technical aspects of the verification including the difficulties in the facial recognition of such a varied group of children. Moreover, we include a comparison to current therapies and detail the ethical issues taken into account.

## 2. SONRIE: Virtual Environment for Oral-Facial Intervention

At present, new technologies provide opportunities for the rehabilitation of children with different neurological disorders [23,24,26,27,28,29,30]. Studies about Virtual Reality systems have good results for clinical use in pediatric rehabilitation, especially in CP [1]. In fact, researches focused on using VR system in CP interventions aimed to prove effectiveness of VR on the body structures and functions, activity and participation according to components in the International Classification of Functioning, Disability and Health [1]. Moreover, conventional rehabilitation programs are shorter and less intensive to guarantee optimal therapeutic results. They cannot satisfactorily increase the motivation of the patient or support activity participation [30]. Many studies have shown that the motivation of patients play a critical role in treatment results [24,26,30]. Hence, virtual environments can also provide more intriguing and competitive conditions, by increasing the motivation of the patient and ensuring active participation, so that less time is used to regain motor skills [1,31].

SONRIE detects oral-facial difficulties for children with CP between the ages of 4 and 12 by using a virtual environment. SONRIE uses the 360 Kinect sensor and is based on games so it is a low-cost solution. During the developmental years, a child spends a large part of his or her time playing. It is the means and the end through which the children receive the challenges that will enable them to reach the different developmental milestones, thanks to what they can learn through play. The importance of the need to help children with CP to play, whether it is by adapting and modifying games to enable their participation, creating games especially for them, or re-training the abilities that will help them participate in each game. In this case, SONRIE adapts accurately to this theoretical rationale, since it invites children to play within a game-oriented environment, which will induce responses from them that could contribute to the re-training of the oral-facial musculature. In addition, it provides an opportunity for play for children who, due to their pathology, face limitations in the type and amount of games in which they can participate in.

It is necessary to consider the developmental age of the child with CP, to adjust the demands of the game to his or her characteristics, and ensure that the game provides the right challenge to enable learning, inclusion, and the acquisition of different types of abilities. Taking into account the information provided by the survey on disability and personal autonomy presented by the Spanish National Statistics Institute (2008) [32]: over 97% of children aged between 6 and 15 had some sort of disability between 2007/2008. Therefore, it is especially relevant that SONRIE System are going to be applied in schools, thus enabling a greater number of affected children to have access to it, and allowing it to be used in a controlled manner by educators that work with these children thus aiding motivation in these groups of children.

Children with CP have difficulty controlling movement, muscle tone and posture, which evidently includes the muscles of the face, necessary for non-verbal expression and even the movements necessary for eating and swallowing. In fact, in the first 12 months of life, more than 90% had clinically significant oral motor dysfunction. In 60% of the children, severe feeding problems preceded the diagnosis of CP [33]. After the first year of life, these children present difficulties when they want to expressing emotions, as well as communicating, which may involve, that they have having social interaction problems.

The mobility of these muscles can be impaired in different ways, one of which is that they produce movements known as “block” or not dissociated. These muscles will act in a non-dissociated way, because the child does not possess the capability of making one muscle independent from the other, causing his or her face to be expressionless [34].

Another difficulty appears when they cannot move a specific muscle (for example, the super ciliary muscle, for an angry expression). To achieve this muscle mobility, their body has learned to compensate by using other facial muscles to perform the function of that muscle. The alteration of muscle tone that these children present is usually mixed, which means that some muscles are spastic (the muscles remain permanently contracted), while others are hypotonic (with diminished muscle tone), normal, or fluctuate from one state to another. When the child is asked to perform a muscle movement in imitation, he or she may sometimes perform it correctly and other times be incapable of doing so. Hence, SONRIE detects if the child makes a compensation with another facial muscle for performing a particular movement, which is something difficult to detect for the therapist only by observing the movement. Besides, it may be useful to aid the neuro-rehabilitation of the oral-facial musculature in children.

SONRIE stores information about other muscles used to compensate the movement and the time spent in the exercise, as well as the ability or inability to perform the exercise correctly. Consequently, it is considered that the movement is right if it is made within the time limit, if the facial muscles responsible for movement are activated and if there is no compensation with other muscles.

The facial musculature helps us to communicate, it also participates in eating and basic oral-facial functions, such as suction, chewing, swallowing, *etc*. Therefore, the muscular impairment will also affect the performance of these functions. The different games of the SONRIE System allow the user to first explore, and then work on the muscles involved in each of the proposed gestures.

Future studies will verify if SONRIE is a serious game able to achieve motor learning. As noted by Sanchez-Cabeza *et al.* repetition of movements enable the development of new learning and new movement schemes in the brain. Furthermore, it allows the child to be an active part of the process, which contributes to an improved self-esteem and personal satisfaction, as well as better motor learning and greater compliance with treatment [35].

## 3. SONRIE Framework Development

**Figure 1:**
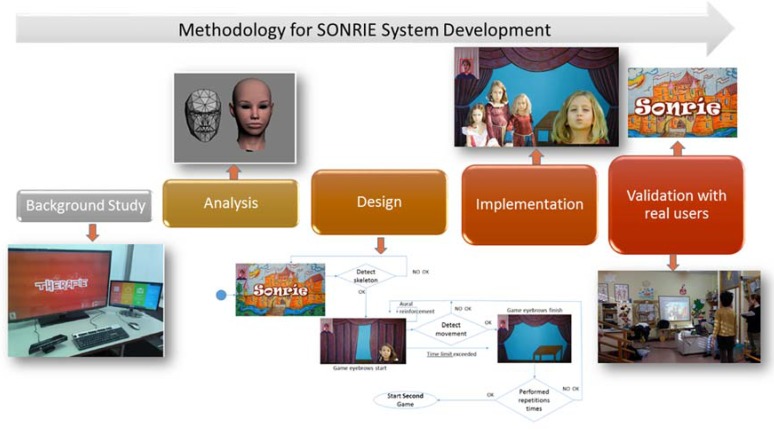
Work methodology used for the construction of SONRIE.

In order to create any information system, it is necessary to have the experience of different types of actors: final users, experts, analysts, developers; moreover, it is essential to design a methodology that covers the stages required to provide the solution—an information system—within the estimated timeframe, and covering the requirements defined. Figure 1 presents the general methodology, which covers the construction phases for SONRIE System development. Furthermore, it is important to indicate that SONRIE System is fully developed, now we are working in the validation stage with children in a real environment over 9 months.

In the Background Study stage, we analyzed the difficulties faced by these children when performing movements that involve the oral-facial musculature. Furthermore, we worked on the study of possible technological solutions with the objective of achieving a neuro-rehabilitation process for these children. Their findings in that moment were that, to this moment, there had been no specific work done on the oral-facial region, and given its implications on the occupational performance of these children, we decided on this musculature as the target area. After that, we decided that: SONRIE uses Microsoft’s Kinect sensor connected to a PC, with an integrated database and video instructions to form the video game. Kinect is a webcam-style add-on for the Windows operating system, which provides a natural user interface (NUI) allowing users to interact intuitively and without any intermediary device, such as a game controller.

The child and the Kinect sensor will be situated in the following positions:

- Child: placed at a distance of 140 cm from the Kinect sensor, standing upright and looking at it.
- Kinect: the elevation above ground is the difference between a child’s height and 80 cm/31.5 inches.

The closer the positions are to these values, the greater the accuracy of the SONRIE system is.

**Figure 2:**
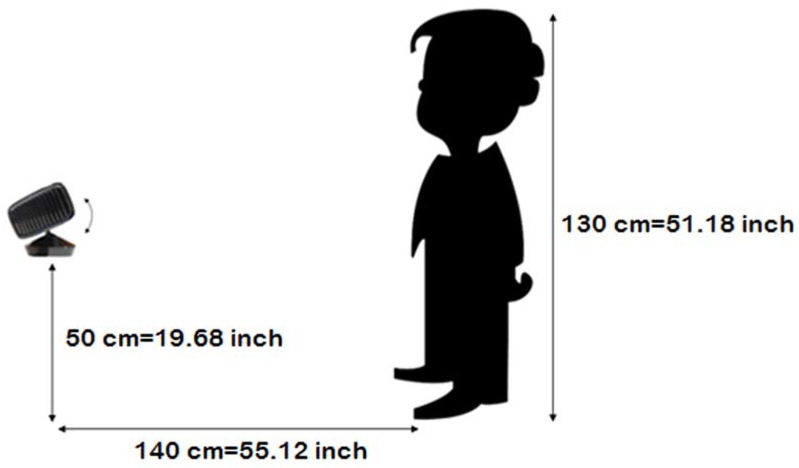
SONRIE game environment configuration.

Figure 2 shows an example: a child 130 cm/51.18 inches tall, who uses the SONRIE system, will be placed at a distance of 140 cm from the sensor, and the sensor should be placed at a height of 50 cm/19.68 inches (130 cm/51.18 inch − 80 cm/31.5 inch = 50 cm/19.68 inch).

Additionally, to make the system more intuitive, a red square appears in the upper left corner of the SONRIE game that indicates where the child’s head should be positioned.

Over 40 facial muscles control our facial expressions, which allow us to show our mood or our current emotions. Besides, they allow human beings to perform one of our most vital functions: eating. Due to the amount of muscles involved, and the variety of their functions, the human being can learn to independently move each of them. However, children with CP are not capable of doing this. For example, when trying to furrow the brow, they can raise one of the corners of their mouth, performing this movement involuntarily, and without getting their facial muscles to move independently. The main difficulties derived from their brain injuries [36], in this area, are: suctioning, communication, imitating facial expressions, involuntarily moving the tongue, babbling and drooling.

The identification of emotions in the face implies gathering information associated with the shape of the eyes, nose and mouth, the location of the apexes, wrinkles, lines and bulges. Estimates, such as age, could also be inferred from the methods employed to detect emotions, if attention is focused on lines or the skin’s luminosity. In the case of children with CP, it is important to get them to execute the movement when they want to, rather than involuntarily. These children face the problem of believing that certain movements are performed correctly, when in reality, they are executing them incorrectly. Because of this, a facial expression recognition motor is needed, to ensure that the movements performed by the children are adequate and offer feedback to the child using the system at that moment.

The last task to perform during this stage consisted of determining four movements as part of the facial movements proposed by SONRIE for children with CP: raising both eyebrows, blowing, kissing and smiling. The occupational therapist decided to create activities in which joining the simplest facial movements. These games achieved a process of detection and treatment of children with CP that not only have motor problems but also visual, cognitive or sensory perception disorders, hearing and learning difficulties.

**Figure 3:**
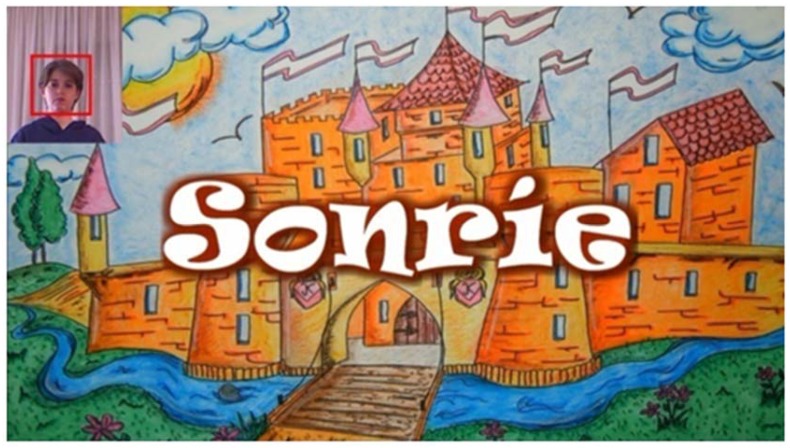
Game theme SONRIE.

All the games of SONRIE can be configured to have different levels of difficulty. The therapist can decide the number of repetitions and the time limit by game. Moreover, with the aim of making the SONRIE games more attractive for children between 4 and 12 years of age, we chose a medieval theme (Figure 3).

**Figure 4:**
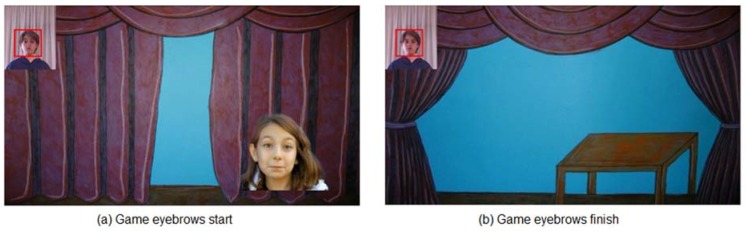
Game scenarios SONRIE. Eyebrow raising game.

SONRIE’s common theme is a game in which the child must overcome several tests. When the test is complete, he or she may advance to the next screen (Figure 4).

Each of these tests is an exercise that will prompt the four movements that the team considered basic for a child with CP.

Furthermore, the development team recorded videos in which three girls (functioning as avatars) indicated to the player how he or she had to perform each of the exercises. We used the Face Tracking Software Development Kit (SDK) for recognizing each of the movements to be detected. It can be used as marker to track human faces with the Kinect camera attached to a PC. The face tracking engine computes 3D positions of semantic facial feature points as well as a 3D head pose. Face Tracking SDK results are also expressed in terms of weights of six Action Units and 11 Shape Units, which are a subset of what is defined in the CANDIDE-3 model [37]. CANDIDE is a parameterized face mask specifically developed for model-based coding of human faces. Its low number of polygons (approximately 100) allows fast reconstruction with moderate computing power. CANDIDE is controlled by global and local Action Units (AUs). The local Action Units control the mimics of the face so that different expressions can be obtained. For example, we decided to use the AU2—Lip Stretcher to detect the blowing movement, as the lip stretchers records the shape of the mouth using dots around the mouth. In AU2, the possible movement, is expressed with a threshold values between −1 and 1 (0 is associated with a neutral expression; 1 indicates that the lips are stretched; and −1 indicates that the lips are totally together and rounded). We choose the value −0.7 at the threshold to indicate that the child is blowing, since −1 indicates that the lips are completely rounded, and the blow has not rounded.

The physical therapist, who works with the child, can configure the game according to the needs and progress of each child, in order to carry out an effective therapy. To achieve this, Table 1 shows that it is necessary to persistently store some child-game configurations. For instance, the therapist configures the number of repetitions for each game and the time limit needed to carry out the game.

It is useful to know the results of running the games of each child. In Table 1 called “child performs game”, SONRIE persistently stores information about the child game execution, with data about if the child has executed or not the movement, the time it took and if the child needed to use other muscles to perform the movement.

**Figure 5:**
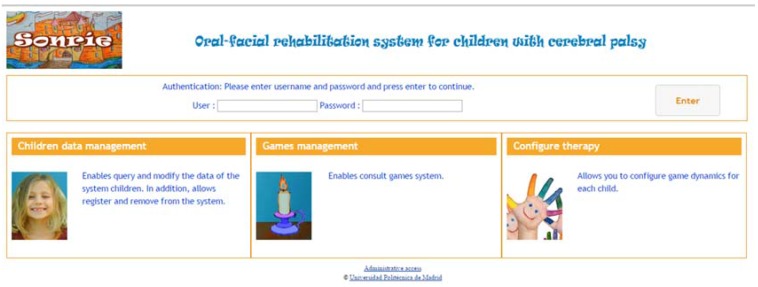
SONRIE Web based framework.

Thus, when the gaming platform starts executing, the stored values associated with each child, offering an adequate dynamic game, and, when finished, the results are stored for later review by a therapist. This work culminated in the completion of the system’s first prototype, which included the four aforementioned games and a Web Based Framework which allows the therapist to work with the child information and to configure games for the children with different possibilities to adjust the therapy for the child (Figure 5 shows the access interface for SONRIE Framework).

The next section explains the general function of the SONRIE System and presents the structure that decides when each of the games should appear.

## 4. SONRIE System Function

Play motivates children and makes them want to participate in a particular activity, as long as it is designed within the parameters that regulate games. Additionally, if it is interactive and technological, its capacity to motivate children increases, and adhesion to treatment improves. The following games cover each of the movements in a simple, guided way, using a motivating interface for the child.

### 4.1. Start and Eyebrow Raising Game

When the game begins, the introduction screen appears (shown in Figure 3); next, after the player’s skeleton has been detected, the eyebrow raising game begins to run.

This game will be running until movement is detected, or the time limit for execution of the exercise has been reached. Figure 3 shows the start and end interfaces of the eyebrow raising game. As we will show below, we designed starting and ending screens that are stimulating for the child for each of the games.

**Figure 6:**
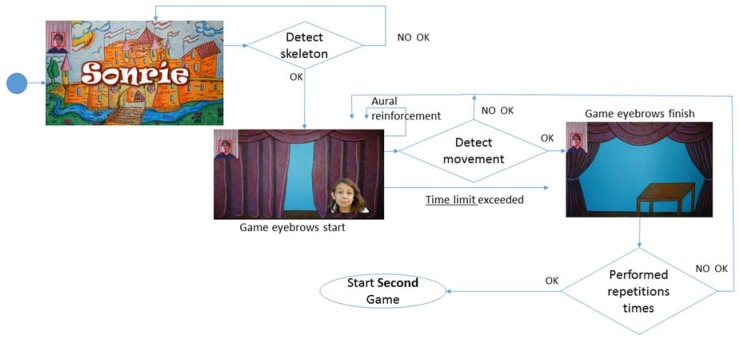
Activity diagram: Start game and eyebrows rise.

If this screen remains after the half of time limit, because the child has not yet performed the movement, a sound will be heard to give the child positive reinforcement (see Figure 6).

Once the gesture is considered appropriate, the number of times the game has been played is counted. If it is the first or second time, the eyebrow video will play again, but if the count is higher, the decision will be made to continue to the next game. In other words, each game must be played the times as repetitions valued, with a time limit to perform each corresponding exercise (see Table 1).

### 4.2. Blowing and Kissing Games

**Figure 7:**
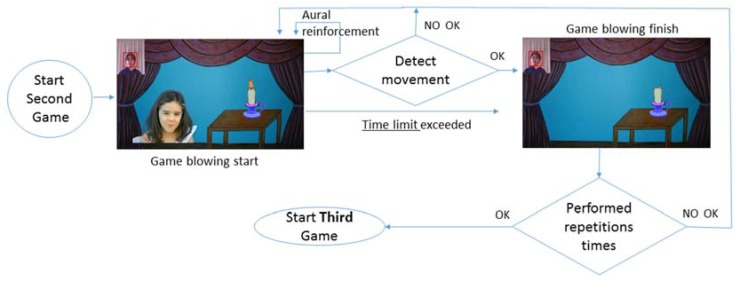
Activity diagram: blowing game.

**Figure 8:**
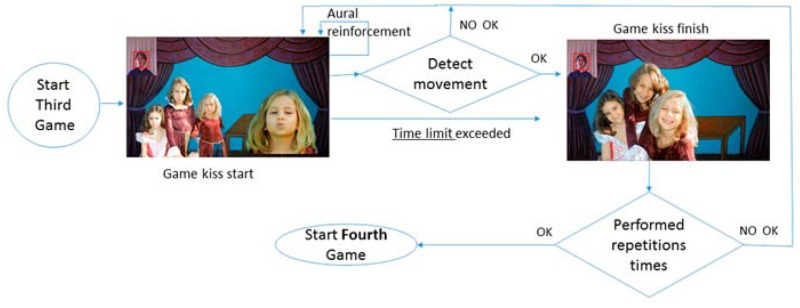
Activity diagram: kissing games.

The blowing (Figure 7) and kissing (Figure 8) games follow the same procedures as the previous one: waiting until the movement is recognized or the time limit reached, to continue to the final video for each game.

After this, the counter is reviewed to see if the same game is repeated or the next one can begin.

### 4.3. Smiling Game and End of the Game

**Figure 9:**
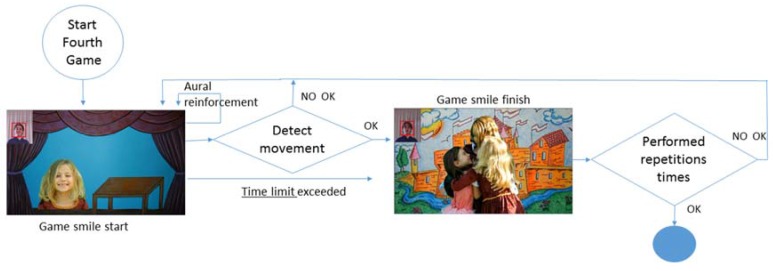
Activity diagram: smiling game.

The smiling game (Figure 9) begins after the kissing game (Figure 8), but unlike the previous games, it does not have an end video that signals that the exercise has been performed correctly; instead, it enters a loop until it has been performed correctly (the gesture is recognized or the time limit has been reached) repetition times, after which it shows way to the video that indicates the end of the game.

**Figure 10:**
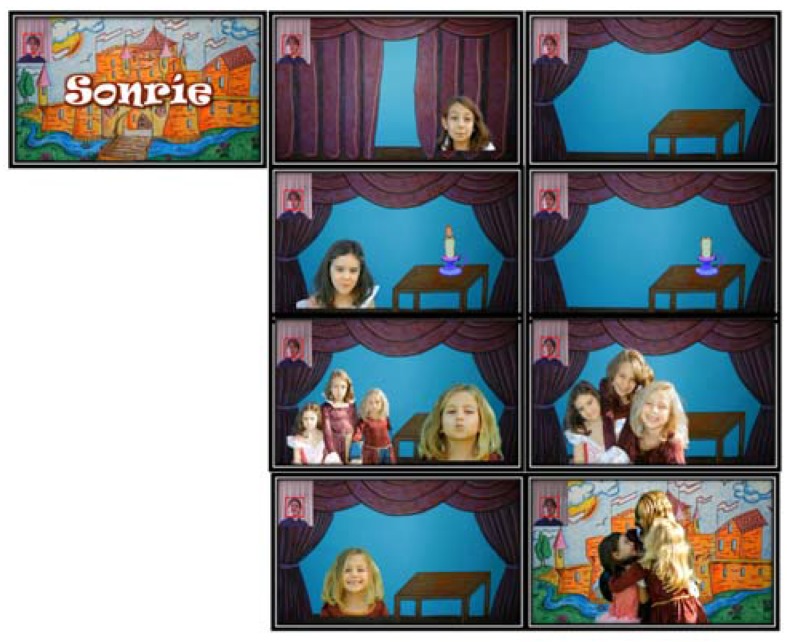
Interaction screens with different games to motivate children.

We summarize the SONRIE functionality in Figure 10, which shows all the screenshots of the SONRIE game. The SONRIE serious game and its framework (Figure 5) have been developed by a multidisciplinary team of professionals. The game’s design has considered the experience gained by occupational therapist while working with children with special needs.

### 4.4. The General Facial Recognition Algorithm for Obtaining the Accurately for Each Game (Numerical Value by Game)

During the execution of each game in SONRIE, the child obtains a numerical value (between −1 and 300) per game, this number indicates the precision and the execution speed of the facial movement supported by SONRIE for the child with CP, or the child with typical development.

Some possible values for the facial recognition algorithm are:

−10: the child performs the opposite movement that is being proposed by SONRIE.
- Values between 0 and 99: the child makes a correct facial movement, nevertheless he/she does not reach the minimum threshold which measures the precision of the facial movement execution.
- Values between 100 and 300: the child carries out successfully facial movement supported by SONRIE serious game. The return value increases depending on the seconds needed and the facial movement precision.

## 5. Verification Stage: Children with Typical Development

Before beginning the experimentation process with children with CP, we evaluated a control group consisting of 7 children with normal development who were 4 to 10 years old. We configured a time limit of 15 s for executing the facial movement repeated three times per child. Children with normal development did not have any disorder, therefore they were able to carry out all the games with precision and execution speed values between 66 and 269. These values were obtained applying the algorithm summarized in the above subsection. Hence, we could verify that all the SONRIE’s games run correctly with healthy children. This verification stage has allowed to calibrate each game to ensure that each movement was detected correctly by SONRIE. Moreover, we evaluated that the facial recognition algorithm works satisfactory. Table 2 shows the average values obtained for each game in tests with healthy children.

**Figure 11:**
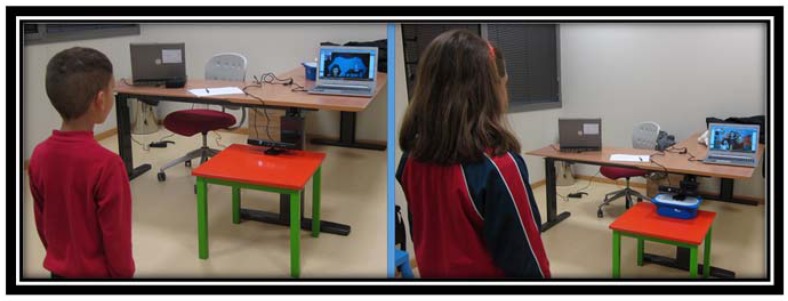
Verification Stage with typical development children.

Table 2 summarizes that healthy children got good scores on average performance on all of the games (95 the average value for the blow game and 173 the average value for the eyebrow game). Figure 11 shows two images of the tests where the Kinect sensor is connected to the computer in which SONRIE serious game is running.

**Figure 12:**
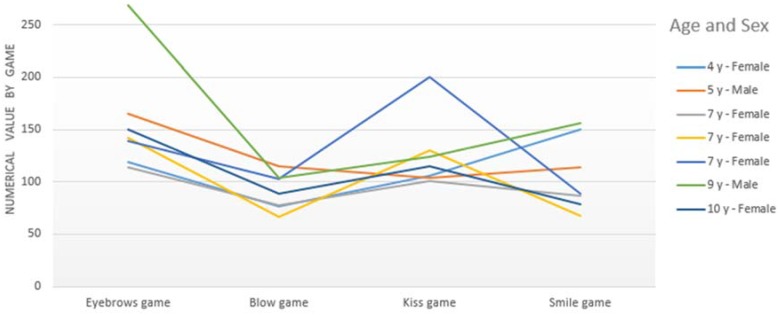
Results obtained by children with typical development.

Figure 12 shows that healthy children perform similarly in the execution of each SONRIE game. The game “Smile” is the most irregular in its execution. In fact, this game obtained values between 67 and 159 corresponding to the precision in which the healthy child performs this movement. In our opinion, this game requires greater precision in its execution to be correctly detected by the Kinect sensor. The “Smile” is the most complex of all of the movements implemented, having many variations.

Furthermore, the green line (see Figure 12) shows the results obtained by a 9 year-old boy who was very focused during the test. As a consequence, he obtained the best results in almost all of the games.

Figure 12 shows the best score for each healthy child. His/her scores were obtained by taking into account the best score in the performance of each game.

## 6. Validation Stage: Children with CP

After the SONRIE verification stage carried out with healthy children, two experimental tests took place, both in a real environment: an integration school for children with CP. Before the beginning of the validation at Bellas Vistas School, we have into account ethical issues to consider in an intervention with a very fragile population: this study was approved by the Ethics Committee of the Rey Juan Carlos University and was carried out in accordance with the Declaration of Helsinki.

Furthermore, this report shows the protocol for this research which must be properly explained to the parents of the children who participating in the study. Parents must approve of the participation of children in the study, by signing an acceptation letter.

This validation stage took place in two different sessions. We considered it relevant to carry out the tests in an environment that was familiar to the children with CP, and that did not imply a disruption to the routine of these children who participated in the study. The children were accompanied by their physical therapist, who helped them understand the dynamics of the game. In order to verify if the children performed well during the exercises, they were evaluated using a scale of facial gestures evaluation of children with Cerebral Palsy (Likert scale-Table 3), created *ad hoc* by the physical therapist working with the team. This scale was used before and after the verification stage. In this moment children executed all the movements to the school’s physical therapist without the SONRIE system. The paper provides in Table 3 a novel scale for measuring facial movement capacity. Future researches should check correlations with other scales such us the “Nordic orofacial test screening (NOT-S)” and should not be based only on observations [38].

The NOT-S test collects mainly the facial muscle movement involved in each exercise. The clinical application is to improve muscles required for feeding and non-verbal communication [39]. Below, we show the results obtained from the 10 children with CP, all are students at an integration school, who participated in the SONRIE’s validation stage.

**Figure 13:**
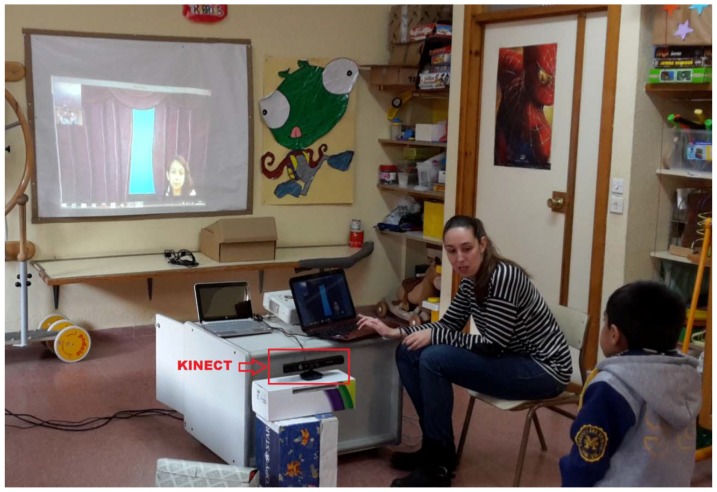
SONRIE validation stage.

The sample chosen for the system’s validation was made up of children with CP, all between 4 and 12 years of age. Figure 13 shows one of the tests with the Kinect connected to the computer on which the SONRIE system is running.

The selection criteria for children were: both genders, within the described age range, with a diagnosis of CP and impairment of the oral-facial musculature. Two verification groups were created randomly, each made up of five children.

The main aim of this validation test was to carry out a test about the way in which children with CP accepted the SONRIE games, and to contrast their results to those obtained in the verification phase. As in the verification phase, we configured a maximum time threshold or time limit (15 s) and the execution of each exercise three times during which the child had to perform the movement. In all the situations, the results of the exercises were stored in a database. In the future it will allow the therapist to specifically configure each game for every child.

**Figure 14:**
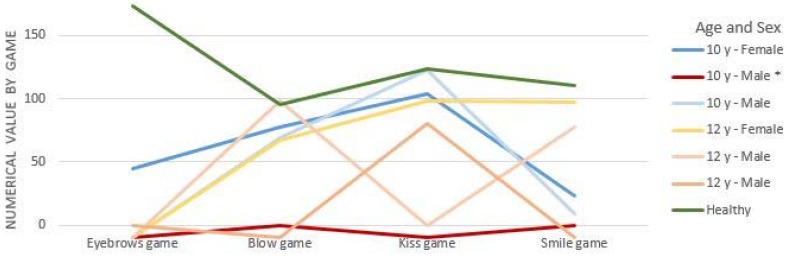
Comparative results obtained by children with CP (10–12 years) and healthy children.

**Figure 15:**
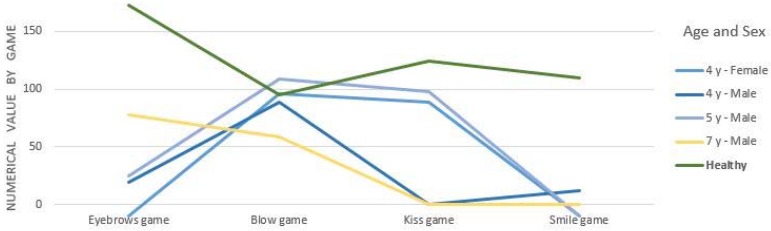
Comparative results obtained by children with CP (4–7 years) and healthy children.

In addition, we obtained interesting results that are shown in Figure 14 and Figure 15. Figure 14 shows a comparison between the scores of children with CP aged between 10 and 12 and the average results obtained by healthy children (line green in Figure 14) evaluated in the verification stage. As shown in Figure 14, the scores of children with CP, regarding the execution of the games, are more irregular between them and for each child. This explains why it is so difficult to find big and homogeneous samples, in addition to the difficulty in generalizing the results of studies conducted in this population [40].

As Figure 14 shows, during the execution of the games, we detected that a 10-year old boy, corresponding to the red line in Figure 14, had a mobility impairment in the musculature of the upper part of the face, a problem that had previously gone undetected. Hence, he could not perform the movements even once. Finally, Figure 15 shows a comparison between the scores of children with CP aged between 4 and 7 with the average results obtained by healthy children (line green in Figure 15) evaluated in the verification stage.

On the one hand, Figure 15 shows that the average scores of all healthy children (green line in Figure 15) are higher than for children with CP. As we might anticipate, it is logical that children without a pathology obtain better scores than children with CP. These normal scores, adjusted by age, will establish normal data for each game when we enlarge the sample of healthy children. On the other hand, Figure 15 shows that the games’ efficient execution is not dependent on age, the data suggests that it is related to the degree of impairment. This hypothesis should be re-evaluated in future studies with the SONRIE serious game.

## 7. Discussion

In this moment, we present a discussion that focuses on three aspects to consider in evaluating a technology as it is presented in this paper. First, technical aspects of the verification: we evaluate the SONRIE functionality testing seven healthy children aged between 4 and 10 years. In these tests, we determined the correct distance between the child and the sensor and we decided that adequate lighting should be provided to optimize the functionality of the Kinect sensor. Furthermore, we adjusted the minimum time length and the number of repetitions every exercise to propose to the school physical therapist as a starting point to begin the assessment in a real environment. As second question, we want to show a comparison to current therapies: CP may affect oral motor skills, leading to speech delay, drooling and difficulties with sucking, swallowing, and chewing [41,42]. Despite the importance of the role of feeding for children with CP and their families, clinical experience shows that oral-motor treatment is not taken care of in the overall rehabilitation of these children. Present and past scientific studies mainly focus on feeding problems. The treatments carried out since 1979 by Borkowska [43] studied the most common problems: sucking, swallowing, breathing, chewing and drooling. Further studies reach different and even contradictory conclusions [44,45,46,47]. Borkowska tested the effect of rehabilitation of the feeding function on the development of visual-motor coordination and speech. He concluded that the training had a significant effect on feeding, and positive influence on the eye-hand coordination if the position was correct. These positive effects are corroborated on the study by Gisel [44] and Sığan [40]. Later, Gisel *et al.* asserted that oral-motor training did not have a significant effect on feeding and growth [45]. Nevertheless, they found that this treatment reduced the feeding problems caused by aspiration.

As to reducing drooling, we have found contradictory results too. While the study of Domaracki and Sisson did not find any treatment benefit [46], positive results were confirmed by the research of Sığan [40] and Yam [47]. Furthermore, Sığan *et al.* discovered in 2013 significant improvements in the treatment group when it comes to chewing, swallowing, drooling and independent feeding.

Therefore, these studies show that there is not a single treatment protocol that is valid and established for the population in this area. Besides, there is not consensus regarding detection systems, screening and/or assessment tools. The scales used to determine the oral-motor dysfunction are mainly focused on feeding problems and they do not evaluate the facial muscles on the whole. Hence, these scales do not take into account those elements related to the role of emotional expression [48].

The clinical tool used to identify the presence of possible neuromuscular problems is the oral mechanism examination [38]. The observation of the feeding function, together with perceptual judgments about strength and joint range of motion are usually the way to obtain information on the performance of feeding. In addition, this kind of information is full of subjectivity; the validity and reliability of these assessments are unknown nowadays. Neither there are normative data that could help distinguish normal from abnormal performance [38].

SONRIE is a proposed technology that provides reliable data on the muscular function of the face as a whole. This research highlights the importance of the detection of oral-facial musculature impairments and represents a starting point to achieve an improvement treatment to be offered to children with CP.

## 8. Conclusions

Using new technologies as a method for detection, intervention and rehabilitation in children with CP is a relatively new field, where we need to continue working to improve these children’s quality of life. For the neuro-rehabilitation of children with CP, it is necessary to have a real environment, where this therapy can be satisfactorily applied. In this environment, the child must feel comfortable and safe. In addition, it is necessary to have the collaboration of a professional that can introduce the child to the game and then measure the child’s degree of improvement as the therapy progresses. Considering the above, the school is the ideal environment to apply the system presented in this research. However, other possible scenarios, such as specialized therapeutic centers and, in the future, the home environment, should not be ruled out.

The SONRIE system can help these children, so they may work on their facial muscles. An improvement in muscular dissociation and an absence of compensations will mean that a child has better facial expressions, and he/she is more independent when eating, which not only contributes to improving his or her quality of life, but also that of his or her family members. The SONRIE system is a highly ecological environment, adapted for children, since it uses interactive games as therapeutic tools. Furthermore, SONRIE serious game allows professionals to create personalized therapies, configure personalized games and cushion the effects produced by CP in children who are treated with this interactive therapy.

We need more than specific knowledge about CP and the oral-facial musculature that intervenes in the performance of different facial movements to satisfactorily develop SONRIE. Furthermore, it is essential to research existing technology and choose, in function of that research, the most adequate device to solve the problem. Lastly, it is necessary to observe these children as they perform the proposed movements, adjusting each of the games developed (smiling, kissing, blowing and raising eyebrows) and adapting them to the specific needs of each child in a real environment. SONRIE will aid the therapy process that children with CP undergo in schools, and will allow professionals to configure personalized therapies. In addition, in the future, we will evaluate whether it is possible to alleviate some of the effects that CP may have on the children treated with this therapy.

The verification and validation results presented in this article justify the need for the better detection and intervention in the oral-facial musculature using a system like SONRIE. This system has proven to be effective because it is easy for professionals to use, motivational for children, and has a low cost. The selection of the game as the element that facilitates therapy was justified theoretically in the section “SONRIE: Virtual Environment for oral-facial rehabilitation”, and it has been proven by the validation process carried out in the school; the games caught the attention of the children and kept the children interested. In future works, we will use SONRIE as a tool for rehabilitation of oral-facial muscles in children with diseases.

## Figures and Tables

**Table 1:** Persistence information about games by children.

Child-Game Configuration			Child Performs Game		
(Primary key)	Id_Game	(FK Game)	(Primary key)	Id_Game	(FK Game)
(Primary key)	Id_Child	(FK Child)	(Primary key)	Id_Child	(FK Child)
(Primary key)	Date		(Primary key)	Date	
	Repetitions			Executed	
	Time limit			Time necessary	
				Offset by other muscles	

**Table 2:** Average values obtained for each game in tests with healthy children.

Description	Eyebrow Game	Blow Game	Kiss Game	Smile Game
Average values obtained by healthy children between 4 and 7 of age	136	92	128	101
Average values obtained by healthy children between 9 and 10 of age	210	97	120	118
Average values obtained by healthy children	173	95	124	110

**Table 3:** Scale of facial movements evaluation of children with Cerebral Palsy.

	1. Never	2. A few times	3. Several times	4. Always
1. The child is able to express surprise or admiration raising eyebrow				
2. The child is able to blow				
3. The child is able to give a kiss				
4. The child is able to smile				
